# Sex differences in the human metabolome

**DOI:** 10.1186/s13293-022-00440-4

**Published:** 2022-06-15

**Authors:** Michele Costanzo, Marianna Caterino, Giovanni Sotgiu, Margherita Ruoppolo, Flavia Franconi, Ilaria Campesi

**Affiliations:** 1 Department of Molecular Medicine and Medical Biotechnology, School of Medicine, University of Naples Federico II, 80131 Naples, Italy; 2 CEINGE – Biotecnologie Avanzate s.c.ar.l., 80145 Naples, Italy; 3 Clinical Epidemiology and Medical Statistics Unit, Department of Medical, Surgical and Experimental Sciences, University of Sassari, 07100 Sassari, Italy; 4 Laboratory of Sex-Gender Medicine, National Institute of Biostructures and Biosystems, 07100 Sassari, Italy; 5 Department of Biomedical Sciences, University of Sassari, 07100 Sassari, Italy

**Keywords:** Metabolomics, Sex differences, Age, Metabolic signature, Liquid biopsy, Biomarkers

## Abstract

**Background:**

The sexual dimorphism represents one of the triggers of the metabolic disparities between the organisms, advising about wild implications in research or diagnostics contexts. Despite the mounting recognition of the importance of sex consideration in the biomedical fields, the identification of male- and female-specific metabolic signatures has not been achieved.

**Main body:**

This review pointed the focus on the metabolic differences related to the sex, evidenced by metabolomics studies performed on healthy populations, with the leading aim of understanding how the sex influences the baseline metabolome. The main shared signatures and the apparent dissimilarities between males and females were extracted and highlighted from the metabolome of the most commonly analyzed biological fluids, such as serum, plasma, and urine. Furthermore, the influence of age and the significant interactions between sex and age have been taken into account.

**Conclusions:**

The recognition of sex patterns in human metabolomics has been defined in diverse biofluids. The detection of sex- and age-related differences in the metabolome of healthy individuals are helpful for translational applications from the bench to the bedside to set targeted diagnostic and prevention approaches in the context of personalized medicine.

**Graphical Abstract:**

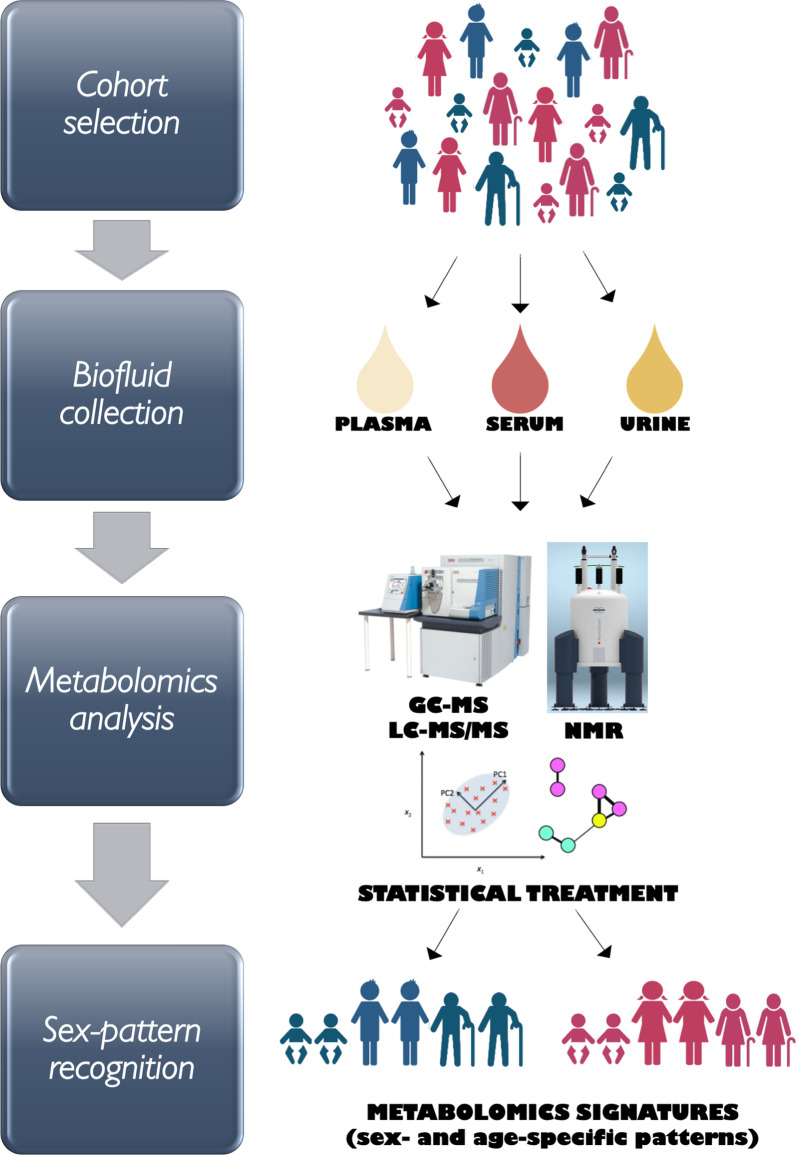

**Supplementary Information:**

The online version contains supplementary material available at 10.1186/s13293-022-00440-4.

## Highlights

- Metabolomics is the gold-standard analytical approach to detect metabolic signatures in human biofluids.
- Analyzing the metabolome at the baseline in healthy individuals is important to identify metabolite signatures to be attributed to one sex or both.
- Differences in the metabolome of biofluids are sex- and age-dependent.
- Sex is not still adequately considered in metabolomics-based investigations.

**Supplementary Information:**

The online version contains supplementary material available at 10.1186/s13293-022-00440-4.

## Introduction

The influence of sexual dimorphism or gender-related dichotomies is gaining mounting interest among scientists in human/animal research or diagnostics fields. The scientific community commonly uses the terms sex and gender as interchangeable, even if they describe specific individual features. Gender specifically outlines the behavioral, psychological, and cultural features of an individual, referring to either social roles (gender role) or personal identification (gender identity). On the other hand, the sex of an individual groups the anatomy of its reproductive system, genitalia, and secondary sex characteristics, defining the elementary biological and dichotomous variables found in most living organisms. Without any social or behavioral consideration, sex represents the trigger of the metabolic disparities between the organisms [1–4].

Metabolomics is the systematic qualitative and quantitative study of the complete set of metabolites in a biological sample. As matter of fact, the metabolome is constituted by several different classes of “small molecules” (80–1200 Da molecular mass) with different chemical and physical properties. The metabolome includes amino acids, peptides, oligonucleotides, sugars, organic acids, ketones, aldehydes, lipids, steroids, alkaloids, xenobiotics, and the small molecules deriving from biosynthetic processes and cell reactions [5]. Metabolomics is able to provide information deriving from healthy and altered metabolic profiles, allowing to understand eventual pathophysiological conditions and the mechanisms that stand behind the metabolic disturbance [6–14].

The variations of the human metabolome might be influenced by numerous factors including methodological issues (i.e., sample collection and technical procedures), genetics, ethnicities, age, lifestyles, hormonal status, and sex [15–18]. The huge amount of data generated by the omics sciences requires particular attention in the steps of data analysis to ensure the accuracy of the results [19, 20]. Thus, the male and female metabolic phenotypes can be investigated through the in-depth analysis of the metabolome in biological fluids [16, 21–25].

In particular, in the current era of the precision medicine, the need to use the so-called “liquid biopsy” like easily accessible body fluids provides great advantages since the collection process is less invasive than tissue collection, and can be reiterated over the time in the processes of follow-up and real-time monitoring of the patients. In addition, the metabolites contained in the liquid biopsies have the power of mirroring multiple biochemical processes and pathways happening in tissues [26–28].

Research evidences endorse the strong differences existing between women and men in the incidence or severity of diseases, the metabolism, and the therapy owing to the different dosage or response to treatments [16, 17, 29–31]. However, many published studies did not stratify the data by sex and did not analyze the data by sex. Even from the current COVID-19 research are perceivable a scarce enrollment of women and a poor stratification of the subjects, and any further analysis based on the sex [32], providing collective results and conclusions valid for both the sexes [33, 34]. Furthermore, the sex differences found in the metabolome vary consistently across different age ranges, from infants to young adults, to the elderly [24, 35–38], underlining the importance of age in determining the sex differences. Finally, it is important to underline that the results based on one-sex analyses alone are not sufficient for drawing general conclusions to be extended to the whole population [39].

Therefore, in this article, the scientific literature was systematically reviewed to evaluate similarities and differences between the human male and female metabolomes of diverse biological fluids (plasma, serum, and urine). Due to the noteworthy technological innovations in metabolomics, the collection of studies published during the last 15 years of research would provide a step forward to understand how sex and age differentially influence the baseline metabolome in healthy individuals.

## Materials and methods

The systematic review of the literature selected in this manuscript was carried out by following the guidelines of the Preferred Reporting Items for Systematic Reviews and Meta-Analyses (PRISMA) system [40, 41].

### Search strategy

The search of literature articles was performed in September 2021, using the MEDLINE/PubMed and ISI Web of Knowledge databases. The search was focused on three main fields/areas that included: (1) metabolomics, (2) sex/gender, (3) healthy populations. In particular, the search was conducted in both the databases using a combination of MeSH terms and variation terms for the above-mentioned fields, using the Boolean search operators “AND” and “OR”. The performed search was set as follows: “(metabolomics OR metabolome) AND (healthy individuals OR control OR sane OR children OR adults) AND (sex OR gender OR sex difference)”. The search looked at the articles published from 2005 to 2021.

### Inclusion and exclusion criteria

The published studies selected by the search were first screened according to the information included in the title and the abstract. Two authors have performed this kind of work independently and then the results were matched. Then, the full-text version of the pertinent articles was taken into account for further consideration. The studies responding to the following criteria were considered: (i) human subjects as total or part of the samples analyzed; (ii) studies including only healthy individuals (or with the main focus on them); (iii) metabolomic analyses (by both NMR and MS techniques) performed on any biological matrix; (iv) articles with main focus on sex-related differences. Analyses performed on only one or few metabolites, on only one sex, lipidomics-based research, methods not based on MS or NMR technologies, articles including disease-state individuals or animal models or in vitro systems, non-English articles, review articles, book chapters, and editorials were excluded. Duplicate publications were deleted.

### Data extraction

The data from the selected studies were extracted and reported, considering: the name of the first author, year of publication, country or ethnicity of the individuals, and the main characteristics of the subjects studied (such as sample size, sex/gender, age), the biological matrices used, and the analytical platforms employed for metabolomics analyses, including details of the downstream statistic tests.

### Studies quality assessment

The quality assessment of the selected studies was performed by two authors independently, using a scoring system specifically created for this analysis (Additional file 1: Table S1). Then, the results were matched and compared for agreement. The studies were classified according to the scores as excellent (11–9), good (6–8), fair (5–4), and poor (< 4). Table 1 summarizes the variables of the score setting used to assess the quality of metabolomics data, according to the experimental design (considering the size and age stratification of the populations), the methodology (including the metabolomic platforms, the statistical analyses, and eventual validation experiments), and the novelty of the research. A score equal to or higher than 6 was considered a minimum for inclusion in the review. Few articles that were difficult to integrate or poorly focused on the main aim of this review were excluded despite the good attributed score.

**Table 1:** Classification of the 32 eligible studies selected for the review according to the score system

First author and year	Experimental design		Methodology			Novelty	Final score	Classification
	N of subjects (per sex)	Age stratification	Analytical platform	Statistical support	Validation			
Chekmeneva E. 2018 [65]	2	1	3	2	1	1	10	Excellent
Dunn W. B. 2014 [38]	2	2	3	2	0	1	10	Excellent
Lawton K. A. 2008 [55]	2	2	3	1	0	1	9	Excellent
Saito K. 2016 [58]	1	2	3	2	0	1	9	Excellent
Andraos S. 2021 [64]	2	2	2	1	0	1	8	Good
Caterino M. 2021 [36]	2	1	2	2	0	1	8	Good
Lau C.-H. E. 2018 [54]	2	1	3	2	0	0	8	Good
Rist M. J. 2017 [56]	2	0	3	2	0	1	8	Good
Ruoppolo M. 2015 [57]	2	1	2	2	0	1	8	Good
Trabado S. 2017 [62]	2	1	2	2	0	1	8	Good
Zaura E. 2017 [49]	2	1	2	2	0	1	8	Good
Bell J. A. 2021 [48]	2	2	1	1	0	1	7	Good
Caterino M. 2020 [35]	2	2	1	1	0	1	7	Good
De Paepe E. 2018 [66]	0	1	2	2	1	1	7	Good
Jovè M. 2016 [68]	2	0	2	2	0	1	7	Good
Liang Q. 2015 [51]	0	1	2	2	1	1	7	Good
Mittelstrass K. 2011 [24]	2	0	2	2	0	1	7	Good
Scalabre A. 2017 [59]	2	2	1	2	0	0	7	Good
Thévenot E. A. 2015 [61]	2	1	2	2	0	0	7	Good
Yu Z. 2012 [53]	2	0	2	2	0	1	7	Good
Fan S. 2018 [67]	2	0	1	2	0	1	6	Good
Gallart-Ayala H. 2018 [52]	0	1	2	1	1	1	6	Good
Li Z. 2018 [50]	2	1	2	1	0	0	6	Good
Ruoppolo M. 2014 [15]	2	1	2	1	0	0	6	Good
Slupsky C. M. 2007 [60]	2	0	1	2	0	1	6	Good
Tsoukalas D. 2019 [47]	2	2	1	1	0	0	6	Good
Vignoli A. 2018 [63]	2	1	1	2	0	0	6	Good
Das M. K. 2014 [46]	0	1	1	2	0	1	5	Fair
Hirschel J. 2020 [45]	2	0	2	1	0	0	5	Fair
Jarrell Z. R. 2020 [44]	2	0	2	1	0	0	5	Fair
Reavis Z. W. 2021 [42]	2	1	0	2	0	0	5	Fair
Takeda I. 2009 [43]	1	0	1	2	0	1	5	Fair

## Results

### Literature search and selection process

**Fig. 1:**
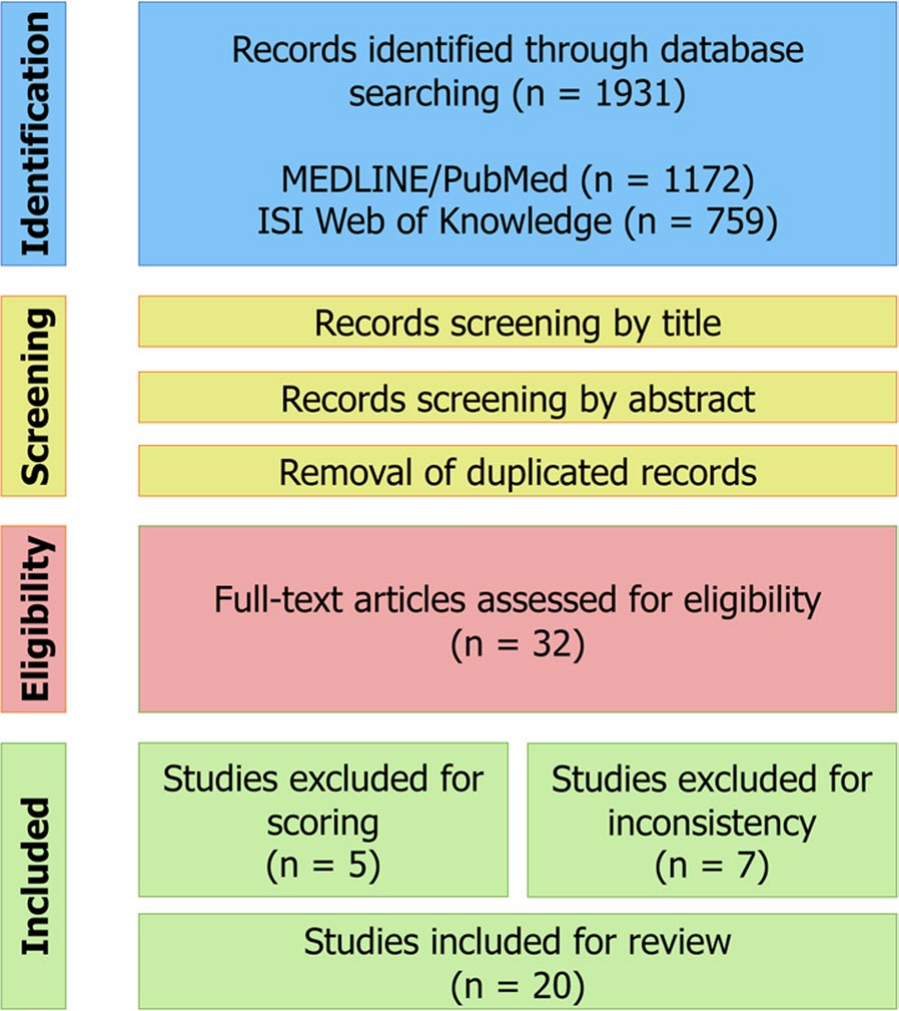
Schematic flow diagram of the entire review process

The selection process of the articles searched in the literature is resumed in the flow diagram (Fig. 1). The literature search was performed using the MEDLINE/PubMed and ISI Web of Knowledge databases, leading to the identification of 1172 and 759 research records, respectively. During the screening process, the results from both the databases were merged, deleting the duplicate publications, and 32 articles were selected for the eligibility, undergoing full-text examination. Table 1 globally reports the scores attributed to each of the 32 studies according to pre-established criteria concerning the experimental design, methodology, and novelty of the research.

After full-text examination, 5 out of 32 studies [42–46] were excluded because the quality score was below 6. Despite the good scores, other 7 articles [47–53] were excluded because they were considered dispersive, or poorly focused on the main aim of this review work, which is to underline sex differences.

Finally, 20 articles [11, 18, 24, 25, 27, 43–57] were selected to be included in the review and summarized in Table 2, reporting the main characteristics retrieved from each manuscript. The 20 selected papers endorse different metabolomic approaches and different matrices, highlighting statistically significant sex-related differences in the plasma, serum, or urine metabolome of healthy patients. In addition, among them, 19 studies reported the age of the participants, with highly variable ranges going from 2 days to 100 years.

**Table 2:** Summary of the characteristics of the 20 studies included in the review

First author and year	Country/ethnicity	Subjects (total n)	Sex (total n)	Age/age groups	Biologic matrix	Analytical platform	Statistics	Quality score
Andraos S. 2021 [64]	Australia	1166 1324	565 M 601 F 174 M 1150 F	11.4 ± 0.5 11.5 ± 0.5 46.2 ± 6.4 43.6 ± 4.8 years (mean ± SD)	Plasma	UHPLC–MS/MS	Linear mixed-effects models Pearson’s correlation	8
Caterino M. 2020 [35]	Italy	291	152 M 139 F	(1–36 months) 1–6 months 7–12 months 13–24 months 25–36 months	Urine	GC–MS	Dunn’s test Kruskal–Wallis test Mann–Whitney U test	7
Caterino M. 2021 [36]	Italy	311	174 M 137 F	48–72 h	DBS	LC–MS/MS	Spearman’s correlation PLS-DA VIP	8
Chekmeneva E. 2018 [65]	USA	132	N. F.	40–59 years	Urine	^1^H NMR DI-nESI-HRMS UPLC–HRMS	PCA OPLS-DA	10
De Paepe E. 2018 [66]	Belgium	10	5 M 5 F	25–41 years	Plasma Urine	UHPLC–MS/MS	CV-ANOVA PCA OPLS-DA	7
Dunn W. B. 2014 [38]	UK	1200	N. F.	(19–81 years) < 40 years 40–49 years 50–64 years > 64 years	Serum	UPLC–MS (+) UPLC–MS (−) GC–MS	ANOVA Kruskal–Wallis test Mann–Whitney U test PLS-DA Random Forests	10
Fan S. 2018 [67]	USA	120	60 M 60 F	N. F.	Urine	GC–MS	Mann–Whitney U test PLS-DA PCA	6
Jovè M. 2016 [68]	Spain	146	68 M 78 F	30–100 years	Plasma	LC–MS/MS (+) LC–MS/MS (−)	ANOVA PCA Student’s *t*-test	7
Lau C.-H. E. 2018 [54]	UK France Spain Lithuania Norway Greece	(1192) 199 157 207 201 229 199	(54.6% M 45.4% F)	6–11 years	Serum Urine	^1^H NMR FIA-MS/MS LC–MS/MS	MWAS with multiple linear regression PCA Pearson’s correlation	8
Lawton K. A. 2008 [55]	Caucasian African-American Hispanic	(269) 61 135 73	(131 M, 138 F) 20M, 41 F 69 M, 66 F 42 M, 31 F	(20–65 years) 20–35 years 36–50 years 51–65 years	Plasma	LC–MS (+) LC–MS (−) GC–MS	ANCOVA ANOVA	9
Mittelstrass K. 2011 [24]	Germany	3080 377	1452 M, 1552 F 197 M, 180 F	32–81 years 55–79 years	Serum	LC–MS/MS	PLS Linear regression Partial correlation analysis	7
Rist M. J. 2017 [56]	Germany	200	99 M 101 F	36–80 years	Plasma Urine	^1^H NMR FIA-MS/MS GC(xGC)-MS HILIC-MS/MS UHPLC–MS/MS	Glmnet PLS SVM	8
Ruoppolo M. 2014 [15]	Italy	76	35 M 41 F	20–45 years	Serum	HPLC LC–MS/MS	SAM Pearson’s correlation Spearman’s correlation	6
Ruoppolo M. 2015 [57]	Italy	3680	1856 M 1824 F	48–72 h	DBS	LC–MS/MS	Mann–Whitney U test Linear regression PCA	8
Saito K. 2016 [58]	Japan	60	15 M 15 F 15 M 15 F	25–35 years 25–35 years 55–64 years 55–65 years	Serum	GC–MS HILIC–MS (−) UHPLC–MS (+) UHPLC–MS (−)	Welch’s two-factor *t*-tests PCA	9
Scalabre A. 2017 [59]	France	90	66 M 24 F	(< 4 mo) < 1 mo 1 months 2 months 3 months	Urine	^1^H NMR	PCA O-PLS CV-ANOVA SRV	7
Slupsky C. M. 2007 [60]	Canada	60	30 M 30 F	16–69 years	Urine	^1^H NMR	ANOVA Mann–Whitney U test PCA PLS-DA	6
Thévenot E. A. 2015 [61]	France	183	100 M 83 F	40.9 ± 10.3 years (mean ± SD)	Urine	UHPLC–MS(/MS)	Mann–Whitney U test O-PLS Spearman’s correlation	7
Trabado S. 2017 [62]	France	800	417 M 383 F	18–86 years 37.6 ± 17.2 (mean ± SD)	Plasma	FIA-MS/MS LC–MS/MS	CV-ANOVA OPLS-DA PCA	8
Vignoli A. 2018 [63]	Italy	844	661 M 183 F	41.0 ± 12.0 years (median ± SD)	Plasma	^1^H NMR	PLS-DA Wilcoxon rank-sum test	6

Most of the categories/terms in the table were ordered alphabetically. Whereas not specified in the papers, the generic LC–MS/MS term was reported for targeted metabolomic analyses; ( +) and (-) refer to positive and negative ionization modes, respectively. Missing information are reported as N.F. (not found). mo = months, y = years, SD = standard deviation

Overall, amino acids (AA), which included canonical AA, their derivatives and analogs, and acylcarnitines (AC) were the most frequently detected molecules in the majority of the works, probably because they are easily quantifiable by standardized targeted LC–MS/MS methods [4, 69–72]. Other metabolites such as OA or carbohydrates are more suitably identified by untargeted GC–MS or NMR-based approaches [73–75]. Consistently with these observations, we decided to not include specifically lipid molecules or lipidomics-based research in the analysis of sex differences and age to dedicate a specific space for lipids in future work.

### Sex differences in the plasma metabolome

The plasma metabolome was analyzed and described in 7 out of 20 papers [55, 56, 62–64, 66, 68]. In particular, the sex differences in the plasma were investigated in the metabolome of healthy adult and old individuals, while one paper [64] focused on both boys and girls and their parents. All the main results are summarized in Table 3.

**Table 3:** Sex differences identified in the plasma metabolome for AA and AC

First author and year		De Paepe E. 2018 [66]	Jovè M. 2016 [68]	Lawton K. A. 2008 [55]	Rist M. J. 2017 [56]	Trabado S. 2017 [62]	Vignoli A. 2018 [63]	Andraos S. 2021 [64]	
Metabolite								Adults	Children
AA and analogues	Alanine							M	F
	Arginine							M	
	Asparagine								M
	Aspartate							M	
	BCAA	M			M	M	M	M	M
	Citrulline							M	M
	Creatine		M	F	F		F		
	Creatinine			M	M	M	M		
	Cysteine							M	M
	Glutamate			M			M	M	
	Glutamine	M					M	M	F
	Glycine	F			F			F	F
	Histidine						M		
	Homocysteine		M						
	Kynurenine		M	M					
	Lysine		F						
	Methionine			M				M	
	*N*-Acetyl-L-Methionine	M							
	Phenylalanine			M			M	M	M
	OH-proline		M					M	F
	Oxoproline			M					
	Proline						M	M	
	Sarcosine				F				
	Serine							F	F
	Threonine								F
	Tyrosine	M					M	M	
	Tryptophan		F	M				M	M
AC	C0			M		M			
	C3					M			
	C5					M			

M, higher levels of the metabolite identified in male individuals; F, higher levels of the metabolite identified in female individuals. Hereon, whereas not specified, the age of the individuals treated in the papers is referred to adults

Overall, in adults, the changes are related to AA (and their derivatives). Most of the authors found higher levels of phenylalanine, glutamate [55, 63, 64], glutamine [63, 64, 66], kynurenine [55, 68], methionine [55, 64], proline [63, 64], and tyrosine [63, 64, 66] in men than in women. Also, the branched-chain amino acids (BCAA) namely valine, leucine, and isoleucine were found more abundant in men [56, 62–64, 66]. On the other hand, 3 articles (2 European and 1 Australian studies) showed that glycine was higher in females than in males [56, 64, 66]. No univocal results were found for tryptophan levels, however, the majority of studies found it increased in Caucasian, African-American, and Australian male adults [55, 64] while in the Hispanic population it was found higher or lower in Hispanic men compared to Hispanic women [55, 68]. Finally, creatinine displayed higher concentrations in men in all four the studies that detected this metabolite as differentially abundant between the sexes [55, 56, 62, 63]. Instead, creatine was identified in three out of the four studies as increased in women [55, 56, 63], whereas in Jovè’s study (see supplementary material of [68]) creatine seems to be upregulated in males.

The sole study performed on parents and their children [64] showed that children and adults present the following concordance: BCAA, citrulline, cysteine, phenylalanine, and tryptophan were higher in males, whereas glycine and serine were higher in females. Opposite variation trends were observed for alanine, glutamine, and OH-proline, being higher in male adults and female children.

Furthermore, Trabado and colleagues’ paper [62] was the only one that evidenced significantly changed plasma AC levels, with a significant increase of the small-chain AC, namely free carnitine (C0), propionylcarnitine (C3), and isovalerylcarnitine (C5) in men versus women [62]. A complete list of the full names for AC is reported in Additional file 1: Table S2.

### Sex differences in the serum metabolome

Seven out of the 20 selected papers with a focus on the serum metabolome detected sex differences and the main findings for serum AA and AC are resumed in Tables 4 and 5, respectively. The high heterogeneity of the results observed was probably related to an effect due to the different ages of the selected cohorts. In particular, 2 publications were focused on cohorts of at-term and premature newborns [36, 57], 1 publication on children [54], and 4 articles on adult cohorts [15, 24, 38, 58].

**Table 4:** Sex differences identified in the serum metabolome for AA

		Adults				Children	Infants	
First author and year		Dunn W. B. 2014 [38]	Mittelstrass K. 2011 [24]	Ruoppolo M. 2014 [15]	Saito K. 2016 [58]	Lau C.-H. E. 2018 [54]	Caterino M. 2021 [36]	Ruoppolo M. 2015 [57]
Metabolite								
AA and analogues	Alanine						F	F
	Arginine		M					
	Asparagine			M	M			
	Aspartate						F	F
	BCAA			M	M			F
	Citrulline			M	M		F	F
	Creatine				F			
	Creatinine	F			M			
	Cysteine				M			
	Glutamine		M	M			F	
	Glutamate			M	M			F
	Glycine		F				F	F
	Histidine			M				
	Lysine				M	F		
	Methionine			M	M			F
	Methionine sulfoxide	F						
	Ornithine		M		M	F		F
	Phenylalanine				M			F
	Proline			M	M			
	Serine		F			F		
	Threonine				M			
	Tryptophan			F	M			
	Tyrosine	F		M	M		F	F
	Putrescine					F		
	*S*-Adenosyl homocysteine				M			
	Serotonin				M	M		
	Caffeine	F						

M, higher levels of the identified metabolite in male individuals; F, higher levels of the identified metabolite in female individuals

**Table 5:** Sex differences identified in the serum metabolome for AC

		Adults				Children	Infants	
First author and year		Dunn W. B. 2014 [38]	Mittelstrass K. 2011 [24]	Ruoppolo M. 2014 [15]	Saito K. 2016 [58]	Lau C.-H. E. 2018 [54]	Caterino M. 2021 [36]	Ruoppolo M. 2015 [57]
Metabolite								
AC	C0			M				M
	C2						M	
	C3				M			
	C4							F
	C5				M			F
	C6						M	
	C8							M
	C10		M		M	F		M
	C12				M	F		M
	C14				M			M
	C16				M			M
	C18		M					
	C5:1				M			F
	C6:1							F
	C8:1							F
	C10:1		M					M
	C10:2							F
	C14:1					F		M
	C14:2					F		
	C16:1					F		M
	C18:1				M		M	M
	C18:2				M		M	M
	C4OH							M
	C14OH							M
	C16OH							M
	C14:1OH					F		
	C18:1OH							M
	C3DC				M			
	C4DC						M	
	C5DC				M			
	C8DC							M
	C10DC							M

M, higher levels of the metabolite identified in male individuals; F, higher levels of the metabolite identified in female individuals

In adults, Ruoppolo (2014) and Saito detected higher levels of methionine, asparagine, proline, tyrosine, glutamate, citrulline, and BCAA in men than in women [15, 58], whereas tryptophan was significantly lower and higher in men in [15] and [58], respectively. Mittelstrass and co-workers [24] found that serine and glycine were more abundant in adult women, whereas glutamine, ornithine, and arginine resulted higher in men. Dunn et al. described high levels of tyrosine in women, as well as methionine sulfoxide and caffeine [38]. In addition, Dunn et al. also found that serum creatinine levels were higher in females, and vice versa Saito detected increased levels for this metabolite in males [58].

In a single study performed on children (6–11 years), Lau et al. [54] discovered higher levels of serine, lysine, ornithine, and putrescine in girls, while serotonin was higher in boys.

Finally, Ruoppolo et al. [57] and Caterino et al. [36] found that both at-term female newborns and premature female infants had higher levels of alanine, aspartate, citrulline, glycine, and tyrosine.

The review of the serum metabolome retrieved some conflicting results on the sex-related distribution of AC, with most of the divergency accounting for a single-sex according to each author. Ruoppolo et al. [15] found that only C0 was higher in men than in women in the follicular phase. All the AC found by Saito [58] and Mittelstrass [24] displayed higher concentrations in male individuals. However, according to Lau et al. [54], the analysis of AC levels in children perceived higher levels in females.

In the cohort of male and female at-term newborns aged 48–72 h, Ruoppolo et al. [57] showed that the adjustment of metabolites levels for newborns' body weight modifies the sex differences displayed without. Body weight adjustment had an amplified effect on alanine, methionine, glycine, valine, and tyrosine that were maintained higher in female babies, whereas it overall reduced or eliminated the sex differences highlighted for AC, with some exceptions. Precisely, the levels of C0 and total esterified carnitine remained higher in male infants also after the adjustment for body weight. The levels of C8, C10, C12, C14, C16, C8DC, and C10DC were significantly higher in male infants, while C4 and C5 in female ones. Additionally, the sex differences in C4OH, C14OH, C16OH, C18:1OH were maintained in male infants, as well as for C10:1, C14:1, C16:1, C18:1, C18:2. On the other hand, the differences concerning C5:1, C6:1, C8:1, and C10:2 levels were accounted to female babies. At last, the second study by Caterino et al. [36] performed on DBS (dried blood spots) agreed with the increased levels of C18:1 and C18:2 in male infants as shown by Ruoppolo et al. [57]. Accordingly, also C2, C6, C18:1, C18:2, C4DC, and the total esterified carnitine were significantly higher in male infants.

### Sex differences in the urine metabolome

Sex differences in the urine metabolome have been found in 9 publications [35, 54, 56, 59–61, 65–67]. In urine samples, the spectrum of the metabolite classes identified, including AA, AC, OA, carbohydrates, and other organic compounds, showed higher heterogeneity if compared to plasma or serum, with expected lower similarities within the selected articles. Tables 6 and 7 resume the sex-pattern recognition for the urinary metabolites described hereon.

**Table 6:** Sex differences identified in the urine metabolome (part 1)

		Adults						Children	Infants
First author and year		Chekmeneva E. 2018 [65]	De Paepe E. 2018 [66]	Fan S. 2018 [67]	Rist M. J. 2017 [56]	Slupsky C. M. 2007 [60]	Thévenot E. A. 2015 [61]	Lau C.-H. E. 2018 [54]	Scalabre A. 2017 [59]
Metabolite									
AA and analogues	2-Aminoadipic acid						F		
	2,6-Diaminopimelic acid		M						
	5-Oxoproline							M	
	Acetyl phenylalanine						F		
	Aminosalicyluric acid						F		
	Creatine	F				F	F		
	Creatinine	M			M	M			
	l-Carnosine		M						
	Glycine			F					
	Isoleucine							F	
	Leucine				M				
	N-Acetylaspartic acid						F		
	Nicotinuric acid						F		
	Proline						M		
	Tyrosine							M	
AC	C0					M			
	C2					M			
	C3						M		
	C5						M		
	C6						M		
	C8						M		
	C9	M							
	C10	M					M		
	C7:1						M		
	C8:1	M					M		
	C9:1						M		
	C10:1	M					M		
	C10:2						M		
	C10:3	M							
	C11:1						M		
	C8OH						M		
	C6:1OH	M							
	C10:2OH	M							
	C5-M-DC						F		
	C6DC						M		

M, higher levels of the metabolite identified in male individuals; F, higher levels of the metabolite identified in female individuals

**Table 7:** Sex differences identified in the urine metabolome (part 2)

		Adults						Children	Infants
First author and year		Chekmeneva E. 2018 [65]	De Paepe E. 2018 [66]	Fan S. 2018 [67]	Rist M. J. 2017 [56]	Slupsky C. M. 2007 [60]	Thévenot E. A. 2015 [61]	Lau C.-H. E. 2018 [54]	Scalabre A. 2017 [59]
Metabolite									
OA	2-Hydroxyglutaric acid			F					
	2-Hydroxyphenyl acetic acid				M				
	3,4-Dihydroxy phenylacetic acid		M						
	4-Hydroxybutyric acid			M					
	4-Deoxythreonic acid				M				
	α-Ketoglutaric acid			M	F		F		
	Capric acid			M					
	Caprylic acid			M					
	Citric acid	F		F	F	F	F		
	Fumaric acid			F		F	F		
	Heptadecanoic acid			M					
	Malic acid			F			F		
	Mevalonic acid						F		
	Oxoglutaric acid						F		
	Pantothenic acid						F		
	Pelargonic acid			M					
	Pyruvic acid						F		
	Stearic acid			M					
	Succinic acid			F					
carbohydrates	D-Fructose		F		F				
	Acetaminophen glucuronide						F		
	Galactonic acid			F					
	Gluconic acid						F		
	Glucuronic acid						F		
	Glyceric acid						F		
	Lyxose			F					
	Maltose			F					
	Pentose						F		
	Threonic acid						F		
	UDP-glucuronic acid			M					
	Xylose			F					
Acylglycines	2-Methylhippuric acid						F		
	3-Methylcrotonyl glycine						F		
	Cinnamoylglycine						F		
	Hippuric acid						F		
	*p*-Hydroxyhippuric acid						F		
	Tiglylglycine						F		
	Valerylglycine						F		
Xen	1-Methylurate	M							
	1-Methylxantine	M							
	Caffeine						F		

M, higher levels of the metabolite identified in male individuals; F, higher levels of the metabolite identified in female individuals. Xen, xenobiotics

Lau et al. showed sex differences in urine from children: isoleucine was increased in females, while 5-oxoproline and tyrosine were found at higher concentrations in males [54]. Caterino et al. discovered several sex differences by analyzing four age groups of infants from 1 to 36 months [35] (Additional file 1: Table S3), while Scalabre and colleagues did not find any sex-specific pattern in the urinary metabolome of infants under the age of 4 months [59].

In the adult population, a small number of AA was found divergent according to sex. In particular, proline [61], leucine [56], l-carnosine, and 2,6-diaminopimelic acid [66] were found increased in males, while higher concentrations of glycine [67] and other non-canonical AA (acetylphenylalanine, 2-aminoadipic acid, *N*-acetylaspartic acid, nicotinuric acid, aminosalicyluric acid) [61] were detected in females. Nonetheless, according to the findings reviewed from plasma and serum metabolomes, also the urinary metabolome appears to be characterized by an increased amount of creatinine in males [56, 60, 65] and creatine in females [60, 61, 65].

Essentially, the analysis of the AC levels has found the agreement of most of the selected papers for their distribution to one sex. In particular, AC molecules were higher in the male urine if compared to the female one [60, 61, 65], with exception of the methylglutarylcarnitine (C5-M-DC), which was higher in females [61].

An even higher heterogeneity was found concerning the sex differences accounting for the class of the OA. Precisely, several authors found in the female urine a specific increase in citric acid [56, 60, 61, 65, 67], fumaric acid [60, 61, 67], malic acid [61, 67], succinic acid, and 2-hydroxyglutaric acid [67], mevalonic acid, pyruvic acid, oxoglutaric acid, and pantothenic acid [61]. A divergence of *α*-ketoglutaric acid levels was accounted both for females [56, 61] and males [67]. Instead, several other OA were detected in the urine of males, such as stearic acid, pelargonic acid, heptadecanoic acid, caprylic acid, capric acid, 4-hydroxybutyric acid [67], 4-deoxythreonic acid, 2-hydroxyphenylacetic acid [56], and 3,4-dihydroxyphenylacetic acid [66].

Concerning the other classes of metabolites, the majority of carbohydrates were found in the urine of female individuals, such as d-fructose [56, 66], xylose, maltose, lyxose, galactonic acid [67], pentose, acetaminophen glucuronide, gluconic acid, glucuronic acid, threonic acid/erythronic acid, glyceric acid [61]. By contrast, UDP-glucuronic acid was present only in male urine [67].

Finally, Thévenot identified an entire group of acylglycines, including hippuric acid, 2-methylhippuric acid, p-hydroxyhippuric acid, tiglylglycine, cinnamoylglycine, 3-methylcrotonylglycine, valerylglycine, as more abundant in female urines, as well as for caffeine [61]. On the other hand, caffeine metabolites, namely 1-methylxanthine and 1-methylurate, were found higher in males by Chekmeneva [65].

### Sex differences are age-dependent

The evaluation of the sex-related differences in the metabolome should take into account another important factor represented by the age, with males and females at different age stages possibly showing age-specific metabolic signatures. Among the selected articles, 11 of them identified age-dependent metabolomics profiles [18, 27, 44, 45, 47, 49–53, 57], but only 8 publications identified an interaction between sex and age or performed analyses for such evaluation [38, 55, 56, 58, 60, 61, 63, 68].

In detail, concerning the analyses of the plasma metabolome, Jovè and colleagues identified a significant separation between the age groups 30–49 years old and the 50–59 years old in females, while the 90–99 years age group was the only one that clustered in men, suggesting for a specific metabolic pattern for nonagenarian male subjects [68]. In addition, a significant interaction between age and sex was shown by Lawton and co-workers: in particular, urea and α-tocopherol significantly increased with the age more in females than in males, while the age-dependent increase in kynurenine and the age-dependent decrease in glycerol-3-phosphate were more pronounced in males [55]. Rist and colleagues observed that phosphate and methionine have generally higher plasma concentrations in younger men. In contrast, ornithine, tyrosine, isocitric acid, glucuronic acid, hippuric acid, choline, and pseudouridine have higher concentrations in older men. For women, the associations of the metabolite profiles with the age were less strong than for men. Ornithine, phenylalanine, glucose, mesoerythritol, glucuronic acid, hippuric acid, choline, and pseudouridine were higher in the plasma of older women, whereas isoleucine, tryptophan, aspartic acid, and malic acid tended to be higher in younger women [56]. Vignoli and colleagues reported greater age-related plasma differences in males than in females: histidine and acetate showed statistically higher concentrations in young males, while alanine and creatine were increased in old males. In females, glucose, glutamine, glycine, tyrosine, and creatine were statistically higher in old females [63].

With regard to the serum, Dunn et al. reported a significant interaction between sex and age for urate, glycerol, hexadecenoic acid, and caffeine [38]. The study by Saito et al. on the serum metabolome of two age-stratified (young and old) Japanese population revealed metabolites and metabolic pathways specifically associated with a single sex in both age ranges. In total, 158 and 130 metabolites showed a statistically significant difference between males and females in the young and old populations, respectively. Specifically, 138 and 113 metabolites were divergent in males in the young (25–35 years) and old (55–64 years) groups, respectively, while only 20 and 17 metabolites were more abundant in the female young (25–35 years) and old (55–65 years) populations, respectively. Moreover, 35 metabolites in males and 41 in females were increased in the young population when compared to the old, while 84 and 81 metabolites were more abundant in males and females, respectively, in the old than the young population [58].

Finally, in the analyses of the urinary metabolome, Rist and co-workers observed glutaric acid, 4-hydroxymandelic acid, N-acetylaspartic acid, creatinine, and sedoheptulose being higher in younger men, whereas hippuric acid, citric acid, 2,5-furandicarboxylic acid, 3-aminoisobutyric acid, and quinolinic acid showed higher concentrations in older men. On the other hand, glutaric acid, succinic acid, N-acetylaspartic acid, tiglylglycine, uracil, 1,5-anhydro-d-sorbitol, sedoheptulose, and creatinine were higher in younger women. By contrast, formic acid, tartaric acid, 2-*O*-methylascorbic acid, 2,5-furandicarboxylic acid, and 4-hydroxyphenyllactic acid were higher in older women [56]. Moreover, according to Slupsky and colleagues, alanine, trigonelline, carnitine, 3-hydroxy-isovalerate, and creatinine were significantly different between the younger and older groups both in the urine of men and women [60], while aspartic acid, oxoglutaric acid, malic acid, methylinosine, dimethylguanosine, aminosalicyluric acid, hydroxyanthranilic acid, 5-hydroxyindolacetic acid, threonic acid, and pyruvic acid showed a significant sex–age interaction in the analyses performed by Thevenot et al. [61].

## Discussion

The metabolomes of serum, plasma, and urine contain a multitude of molecules that can be intrinsic or extrinsic factors, of which most may have clinical relevance as used as biomarkers. Thus, metabolomics is easily included in any experimental design to provide support for disease diagnosis, and verify the severity and eventually the efficacy of treatments with the aim of transferring the metabolomics signatures into the clinical care, especially in the perspective of applying approaches to personalized medicine [76–78].

Here, we decided not to take into account the studies predominantly based on lipidomics investigations. Originally, the lipidome was considered a subcategory of the metabolome, being referred to as “*the full characterization of lipid molecular species and their biological roles with respect to expression of proteins involved in lipid metabolism and function, including gene regulation*” [79]. The huge biochemical complexity of lipids and the initial lack of analytical tools for their large-scale analysis have empowered the technologies behind lipid identification and quantification [79–82]. Thus, next to metabolomics, lipidomics science emerged as an additional discipline, deserving an appropriate space and independent consideration.

Globally, this systematic review attempted at categorizing the abundance of many metabolites to one sex or the other, also considering possible variations related to the age of the analyzed individuals. As demonstrated by the critical selection of only a small number of articles, our work showed that sex is not still adequately considered in metabolomics-based investigations, despite the mounting evidence of its clinical importance for diagnosis, therapy, and outcome [83]. In particular, the low number of eligible manuscripts that consider the baseline metabolome in healthy individuals, in combination with the high heterogeneity of their data owing to the different metabolomics platforms, statistical approach, and population size, have hindered any process of meta-analysis that was initially planned. Thus, we decided in the first instance to rely on the qualitative synthesis of the findings from each study. Nonetheless, the common tendency for each metabolite to be quantitatively more represented in one sex, according to authors' criteria, was considered. This allowed the semi-quantitative recognition of metabolites distribution across the sexes considering individuals within similar age ranges.

Thus, amongst the major sex-discriminatory metabolites, creatine was commonly identified as more abundant in females and creatinine as more abundant in male individuals, in all the biofluids analyzed and regardless of age. The amount of creatine and creatinine are often associated with the diet intake and the nutritional state, albeit creatine is also synthesized in vivo in the liver [84]. Creatine is detectable in multiple tissues, despite its concentrations and distributions vary consistently. Skeletal muscle, heart, and spermatozoa show the highest levels of creatine; an intermediate pool of creatinine is found in the brain, brown adipose tissue, and intestine, while low levels are tracked in lung, spleen, kidney, liver, white adipose tissue, and serum [84–86]. The levels of free creatine in vertebrates depend on the creatine kinase enzyme, which catalyzes the reversible transfer of the phosphate group from ATP to the creatine. The flexibility of creatine kinase in adapting its function according to the physiological state of each tissue is enabled by the energy demand [86]. According to the mounting consideration in the field of biochemistry of the relevance of the creatine/creatine kinase system for energy metabolism, sex differences lean toward an accumulation of creatine in female individuals. Besides a probable increased endogenous synthesis [60] and a lower creatine loss in females [84], this sex pattern might be also associated with the increased levels of circulating creatine kinase in males [87–89], which could diminish the amount of free creatine, enriching the pool of phosphocreatine.

The first step of creatine synthesis takes place in the kidney requiring glycine, methionine, and arginine. The entire glycine molecule is metabolized for the synthesis of creatine but only the methyl group from methionine and the amidino group from arginine are consumed [84]. While the latter amino acids showed from our review a distribution toward the male sex, glycine burden is predominant in females in all the biofluids analyzed, possibly justifying the need for a prompt synthesis of creatine.

The creatine synthesized de novo in the liver, together with the dietary creatine absorbed by the intestine, is transported through the blood to the creatine-requiring tissues, such as the muscle that takes up to 94% of the total creatine, being not able to make it by synthesis. The muscle creatine is non-enzymatically transformed into creatinine at a virtually steady rate (∼2% of the total creatine per day). Then, creatinine by diffusing out of the muscle cells is excreted by glomerular filtration into the urine [86]. Accordingly, we found a tendency of creatinine accumulation in biofluids from male individuals, including urine. Therefore, higher creatinine levels in men are reasonable considering that they are typically endowed with increased muscle mass than women.

Moreover, a robust tendency of BCAA to be distributed in the biofluids of male subjects was evidenced. As for creatinine, the differences in the muscle metabolism, muscle size, and the higher dietary protein intake of men may easily justify this tendency as compared to women [63].

In the mitochondria, the free carnitine can be conjugated to acyl-CoA molecules to produce AC, whose synthesis is sustained by the metabolism of fatty acids, AA, and glucose. Hence, the concentrations of circulating AC are dependent on the dietary regimen and may reflect the contribution of specific organs [90]. The levels of AC in the metabolomes analyzed in this review did not show a sharp sex distribution, despite only few authors found some AC predominantly increased in men biofluids. This could be partially legitimated by higher food intake in men. In addition, since AC variations are markers of metabolic and mitochondrial impairment in inborn errors of metabolism (including fatty acid β-oxidation disorders and organic acidemias, among others) [70, 91], and are associated with increased risk of obesity, type-2 diabetes, and cardiovascular diseases [90, 92, 93], individuals in a good healthy state may not show a significant sexual dimorphism in AC patterns.

Finally, the high heterogeneity of the organic compounds identified by each author, especially in urine, did not allow to track specific sex patterns for the majority of the metabolites here reviewed. Notwithstanding, citric acid levels were more elevated in women, as already accredited in the literature [56, 94–96]. Given that also other Krebs cycle intermediates, namely α-ketoglutaric acid and fumaric acid, showed sex divergency being increased in women, one may conceive of general sex differences in the Krebs cycle route, although the reason behind this explanation remains largely speculative [56].

What is more, here is provided some evidence that sex can highly affect the metabolome of diverse biological fluids in an age-dependent way, as already suggested for some serum or plasma biomarkers [97]. Indeed, from a critical analysis of our review work, the data here discussed present some variability in the metabolome of each biofluid analyzed, which could depend on the different age ranges of the participants. Hence, the sex stratification in every single class of age is highly advisable for the validation of reference values to increase their diagnostic and therapeutic appropriateness.

Moreover, we advise that different ethnicity, geographical localization, microbiome, and lifestyles such as the particular choice of foods or beverages, the smoking habit, and the use of hormonal therapies or other drugs are certainly able to differently influence the distribution of metabolites within both sexes. In fact, it is established that the serum metabolome of women is highly influenced by the sex hormones, including the exogenous hormones derived from oral contraceptives [15] or hormone replacement therapy, as well as the endogenous physiological changes occurring during menopause [98, 99].

In conclusion, sex and age should be strongly considered in the planning and the execution of metabolomic investigations, being such differences among the individuals the result of the influence of genome, transcriptome, and proteome. Thus, cumulative intrinsic and extrinsic factors affect the quantitative changes in the metabolome, differentially influencing the individual response of every single subject. Therefore, all the possible variables influencing the human metabolome should be carefully considered in order to reach an authentic personalized medicine.

## Perspectives and significance

The outreach inspired by this review relies on the important consideration of sex patterns in all the aspects of research in health and disease, including metabolomics. In this review, several manuscripts were screened, but only a small number responded to the need of investigating the sex dichotomies in the metabolome of healthy subjects. Thus, we recognized that sex is not still adequately considered in metabolomics-based investigations. Historically, the female sex has been under-represented in both human and animal experimentations, being considered a confounding variable [83, 100]. This outlook can draw misleading evidences that are not equally applicable to both sexes. Accordingly, the same inaccurate interpretation of the results could follow when male- or female-dominant, and mixed-sex data are analyzed, giving rise to false positives and negatives accounting for one or the other sex, or masking existing differences [39].

Instead, nowadays, the differences between males and females undergo magnification, in order to understand the bases of sex dissimilarities [1]. This gains extreme power when healthy cohorts are analyzed, aiming at dissecting the differences in the metabolome at the baseline, and consequently acting on the bases of such considerations in the medical- or disease-related contexts. Thus, sex consideration should be encouraged in the phases of research planning and actuation, designing studies equally representing both sexes as subjects and eventually performing omics investigations in mixed-sex, and male- and female-separated cohorts [39]. The evidences reviewed in this manuscript do not allow drawing general and specific conclusions to be extended to the whole population. Nonetheless, strong indications were given on the sex-pattern recognition of several metabolites in men or women, which can help outlining the bases of future research.

## Supplementary Information

**Additional file 1: Table S1.** Score setting for quality assessment of metabolomics studies. **Table S2.** A list with the complete names of all the AC described in this review. **Table S3.** Sex differences identified by Caterino et al. [35] in the urinary organic acids of 4 age groups of infants-to-children individuals.

## Data Availability

All data generated or analyzed during this study are included in this published article and its Additional files.

## Abbreviations

ANOVA: Analysis of variance
ANCOVA: Analysis of covariance
CSF: Cerebrospinal fluid
CV-ANOVA: Cross-validated ANOVA
DI: Direct infusion
DBS: Dried blood spot
F: Females
FIA: Flow injection analysis
GC: Gas chromatography
glmnet: Generalized linear model net
HRMS: High-resolution mass spectrometry
HILIC: Hydrophilic interaction liquid chromatography
LC: Liquid chromatography
M: Males
MS: Mass spectrometry
MS/MS: Tandem mass spectrometry
MWAS: Metabolome-wide association study
n-ESI: Nano-electrospray ionization
NMR: Nuclear magnetic resonance
OA: Organic acids
O-PLS: Orthogonal projection to latent structures
OPLS-DA: Orthogonal partial least squares-discriminant analysis
PCA: Principal component analysis
PLS-DA: Partial least square-discriminant analysis
SAM: Significance analysis of microarrays
SD: Standard deviation
SRV: Statistical recoupling of variables
SVM: Support vector machine
U(H)PLC: Ultra (High) performance liquid chromatography
VIP: Variable importance on projection
