# Supplementary material for: Sex differences in the human metabolome
Source: Biol Sex Differ. 2022 Jun 15;13:30. doi: 10.1186/s13293-022-00440-4 (PMC9199320; doi:10.1186/s13293-022-00440-4)
Supplement: Supplementary file 1 — Additional file 1: Table S1. Score setting for quality assessment of metabolomics studies. Table S2. A list with the complete names of all the AC described in this review. Table S3. Sex differences identified by Caterino et al. [35] in the urinary organic acids of 4 age groups of infants-to-children individuals. [file 13293_2022_440_MOESM1_ESM.docx]

**Additional file 1: Table S1.** Score setting for quality assessment of metabolomics studies.

| **Score setting** | | | |
| --- | --- | --- | --- |
| **Article section** | **Maximum score** | **Characteristics** | **Score Attribution** |
| Experimental Design | 2 | Number of subjects (per sex) | 0 🡪 n < 20  1 🡪 20 < n < 40  2 🡪 n > 40 |
|  | 2 | Age Stratification | 0 🡪 Wider ranges or not specified  1 🡪 Narrower ranges  2 🡪 Group-separation analysis |
| Methodology | 3 | Analytical platform | 1 🡪 ^1^H NMR, limited standards  1 🡪 GC-MS, limited standards  2 🡪 LC-MS/MS, extensive standards  3 🡪 Multiple platforms |
|  | 2 | Statistical support | 0 🡪 No statistical support  1 🡪 Univariate statistics and additional normalization or grouping analysis  2 🡪 Univariate and multivariate (PCA, PLS-DA, OPLS-DA, etc.) and other statistics and bioinformatics tools |
|  | 1 | Validation | 0 🡪 No validation experiments  1 🡪 Any experiment to validate one or more metabolite candidates |
| Novelty | 1 |  | 1 🡪 New information in the literature or special focus on sex difference (also consider the year of publication and the size of the study) |
| **Maximum total score** | **11** |  | |

**Additional file 1: Table S2.** A list with the complete names of all the AC described in this review.

C0 = free carnitine

C2 = acetylcarnitine

C3 = propionylcarnitine

C4 = iso-/butyrylcarnitine

C5 = iso-/valerylcarnitine/2-methylbutyrylcarnitine

C6 = hexanoylcarnitine

C8 = octanoylcarnitine

C9 = nonanoylcarnitine

C10 = decanoylcarnitine

C12 = dodecanoylcarnitine

C14 = tetradecanoylcarnitine (myristoylcarnitine)

C16 = hexadecanoylcarnitine (palmitoylcarnitine)

C18 = octadecanoylcarnitine (stearoylcarnitine)

C5:1 = tiglylcarnitine

C6:1 = hexenoylcarnitine

C7:1 = heptenoylcarnitine

C8:1 = octenoylcarnitine

C9:1 = nonenoylcarnitine

C10:1 = decenoylcarnitine

C10:2 = decadienoylcarnitine

C10:3 = decatrienoylcarnitine

C11:1 = undecenoylcarnitine

C14:1 = tetradecenoylcarnitine

C14:2 = tetradecadienoylcarnitine

C16:1 = hexadecenoylcarnitine

C18:1 = octadecenoylcarnitine

C18:2 = octadecadienoylcarnitine

C4OH = hydroxy-butyrylcarnitine

C8OH = hydroxy-octanoylcarnitine

C14OH = hydroxy-tetradecanoylcarnitine (hydroxy-myristoylcarnitine)

C16OH = Hydroxy-hexadecanoylcarnitine (hydroxy-palmitoylcarnitine)

C6:1OH = hydroxy-hexenoylcarnitine

C10:2OH = hydroxy-decadienoylcarnitine

C14:1OH = hydroxy-tetradecenoylcarnitine

C18:1OH = hydroxy-octadecenoylcarnitine

C3DC = malonylcarnitine

C4DC = methylmalonyl-/succinylcarnitine

C5DC = glutarylcarnitine

C5-M-DC = methylglutarylcarnitine

C6DC = adipoylcarnitine

C8DC = suberylcarnitine

C10DC = sebacylcarnitine

**Additional file 1: Table S3.** Sex differences identified by Caterino et al. [35] in the urinary organic acids of 4 age groups of infants-to-children individuals.

| **Caterino et al. (2020)** [35] | Age group 1 (1-6 months) | Age group 2 (7-12 months) | Age group 3 (13-24 months) | Age group 4 (25-36 months) |
| --- | --- | --- | --- | --- |
| **METABOLITE** |  |  |  |  |
| 2-ethyl-3-hydroxy-propionate | F | F | M |  |
| 2-hydroxy-isobutyrate | F |  |  |  |
| 2-hydroxy-glutarate | M |  |  |  |
| 2-methyl-3-hydroxy-butyrate | F | F |  |  |
| 3-hydroxy-isobutyrate | M |  |  |  |
| 3-hydroxy-propionate |  |  |  | F |
| 4-hydroxy-phenylacetate | M |  |  |  |
| 3-methyl-glutaconate |  |  | F | F |
| 3-methyl-glutarate | F |  |  |  |
| α-ketoglutarate |  | F | F | F |
| adipate |  |  | M |  |
| azelate |  |  |  | M |
| citrate |  |  | F |  |
| ethylmalonate | M |  |  |  |
| fumarate | M | F |  |  |
| glutarate | M |  |  |  |
| glycolate |  |  | F |  |
| hippurate | F |  | M |  |
| lactate | M |  |  |  |
| methylsuccinate | F | F |  |  |
| oxalate |  |  | F |  |
| pimelate |  | F |  |  |
| pyruvate |  | F |  | F |
| pyroglutamate | M |  |  |  |
| sebacate | M |  |  |  |
| stearate | F | F |  |  |
| suberate | M |  |  |  |
| succinate | M |  |  |  |
| uracil | F |  |  |  |

M = higher levels of the metabolite identified in male individuals; F = higher levels of the metabolite identified in female individuals.
